# Formulation and Efficacy of Catalase-Loaded Nanoparticles for the Treatment of Neonatal Hypoxic-Ischemic Encephalopathy

**DOI:** 10.3390/pharmaceutics13081131

**Published:** 2021-07-23

**Authors:** Andrea Joseph, Chris W. Nyambura, Danielle Bondurant, Kylie Corry, Denise Beebout, Thomas R. Wood, Jim Pfaendtner, Elizabeth Nance

**Affiliations:** 1 Department of Chemical Engineering, University of Washington, Seattle, WA 98195, USA; ajoseph1@uw.edu (A.J.); cnyambr@uw.edu (C.W.N.); dbond98@uw.edu (D.B.); dbeebout@uw.edu (D.B.); jpfaendt@uw.edu (J.P.); 2 Division of Neonatology, Department of Pediatrics, University of Washington, Seattle, WA 98195, USA; kcorry@uw.edu (K.C.); tommyrw@uw.edu (T.R.W.)

**Keywords:** hypoxia-ischemia, hydrophobic-ion pairing, catalase, nanomedicine, neonatal, molecular dynamics

## Abstract

Neonatal hypoxic-ischemic encephalopathy is the leading cause of permanent brain injury in term newborns and currently has no cure. Catalase, an antioxidant enzyme, is a promising therapeutic due to its ability to scavenge toxic reactive oxygen species and improve tissue oxygen status. However, upon in vivo administration, catalase is subject to a short half-life, rapid proteolytic degradation, immunogenicity, and an inability to penetrate the brain. Polymeric nanoparticles can improve pharmacokinetic properties of therapeutic cargo, although encapsulation of large proteins has been challenging. In this paper, we investigated hydrophobic ion pairing as a technique for increasing the hydrophobicity of catalase and driving its subsequent loading into a poly(lactic-co-glycolic acid)-poly(ethylene glycol) (PLGA-PEG) nanoparticle. We found improved formation of catalase-hydrophobic ion complexes with dextran sulfate (DS) compared to sodium dodecyl sulfate (SDS) or taurocholic acid (TA). Molecular dynamics simulations in a model system demonstrated retention of native protein structure after complexation with DS, but not SDS or TA. Using DS-catalase complexes, we developed catalase-loaded PLGA-PEG nanoparticles and evaluated their efficacy in the Vannucci model of unilateral hypoxic-ischemic brain injury in postnatal day 10 rats. Catalase-loaded nanoparticles retained enzymatic activity for at least 24 h in serum-like conditions, distributed through injured brain tissue, and delivered a significant neuroprotective effect compared to saline and blank nanoparticle controls. These results encourage further investigation of catalase and PLGA-PEG nanoparticle-mediated drug delivery for the treatment of neonatal brain injury.

## 1. Introduction

Neonatal hypoxic-ischemic encephalopathy (HIE) is a devastating neurological condition that affects 1.3–4.7 in 1000 live births in the United States [1,2]. The current standard-of-care, therapeutic hypothermia (TH), is only clinically implemented in cases of moderate or severe HIE and still leaves more than 30% of infants dead or with severe disability [3]. One strategy to improve neonatal outcomes is to use a pharmaceutical agent to complement the neuroprotective mechanisms of TH. For example, erythropoietin (Epo) has neuroprotective anti-inflammatory and anti-oxidant properties and has been successfully translated from the Vannucci model of HIE in rats to non-human primates and is now in clinical trials [4,5]. Another promising therapeutic may be catalase, a large (240 kDa) enzyme that converts the reactive oxygen species (ROS) hydrogen peroxide to water and molecular oxygen. By improving oxygen status, removing ROS, and subsequently suppressing inflammation, catalase has demonstrated efficacy against a range of pathologies including solid tumors [6,7], inflammatory bowel disease [8], and vascular dysfunction [9]. For neurological applications, catalase can attenuate neuroinflammatory and apoptotic pathways in vitro [10,11], but its application in vivo has been limited due to its short half-life, proteolytic degradation, immunogenicity, and inability to cross the blood-brain barrier and penetrate within the brain [12,13].

Nanoparticles serve as vehicles that can improve drug biodistribution and bioavailability. Drug-loaded biodegradable nanoparticles composed of poly(lactic-co-glycolic acid)-poly(ethylene glycol) (PLGA-PEG) have been shown to improve drug solubility, stability, circulation time, release kinetics, and transport to and within the brain parenchyma [14,15]. Enzymes can particularly benefit from nanoparticle encapsulation as the polymer matrix provides protection from immune clearance and systemic degradation [16]. However, the hydrophilic nature of enzymes limits their encapsulation into the hydrophobic core of PLGA-PEG nanoparticles. Hydrophobic ion-pairing (HIP) is a recently-developed technique that increases the lipophilicity of peptides and proteins [17,18]. In this technique, complexes are formed by electrostatic interactions between ionizable groups on the protein and an ion-pairing agent. Complexes are reversible and can dissociate in ionic solutions, but they are also lipophilic due to hydrophobic groups on the ion-pairing agent [19]. HIP has previously been used for PLGA nanoparticle encapsulation of small peptides, antibodies, and proteins as large as bovine serum albumin (BSA, 60 kDa) [19,20,21,22]. Complexation of large enzymes such as catalase, and subsequent nanoparticle formulation and evaluation, has not yet been reported.

In this study, we determine the effects of ion-pairing agent, molar ratio, pH, and buffer ion on catalase HIP complexation efficiency. We use molecular dynamics (MD) simulations to probe the effect of each ion-pairing agent on the protein structure and bring insight into the HIP complexation process. MD simulations can investigate the molecular-scale interactions between enzymes and polymers for drug delivery applications [23,24]. Using an optimized catalase complex, we next develop a PLGA-PEG nanoparticle formulation which provides high catalase activity and protection in degradative conditions. Finally, we assess the efficacy of the catalase-loaded PLGA-PEG nanoparticles in the Vannucci model of HIE in neonatal rats.

## 2. Materials and Methods

### 2.1. Preparation of HIP Complexes

Stocks of each ion-pairing agent were made in DI water: 20 mM dextran sulfate (DS, M_r_ 5000, Millipore Sigma, Burlington, MA, USA), 250 mM sodium dodecyl sulfate (SDS, Millipore Sigma), and 500 mM taurocholic acid (TA, Millipore Sigma). Phosphate buffer (50 mM) was adjusted to pH 4.7 with 0.1 N HCl and then used to dissolve catalase at a 10 mg/mL concentration. Based on molar ratio, an appropriate volume of IP agent (less than 25 μL) was slowly added to the catalase solution, spontaneously forming HIP complexes. The solution was vigorously vortexed for 1 min followed by centrifugation at 12,000 rpm for 15 min at 4 °C. Uncomplexed catalase in the supernatant was measured by bicinchoninic acid (BCA) assay. Pelleted HIP complexes were lyophilized into powder and stored at 4 °C.

The above procedure was modified appropriately for individual experiments: The catalase solution pH was adjusted to 4.2, 5.2, and 7.0 for the pH variation study. Citrate buffer (10 mM) was used instead of phosphate buffer to determine the effect of buffer ion species. For bovine serum albumin (BSA) complexes, BSA (Life Technologies, Carlsbad, CA, USA) was dissolved in citrate buffer at pH 3.7 prior to the addition of IP agent.

### 2.2. Characterization of Catalase Binding Efficiency and Mass by BCA Assay

Binding efficiency was measured indirectly by measuring protein concentration in the initial solution and supernatant using the Pierce BCA Protein Assay Kit (ThermoFisher, Waltham, MA, USA). Following the manufacturer’s instructions, 25 μL of sample was added to 96-well plate in triplicate on ice. After addition of 200 μL BCA assay working reagent (50:1 reagent A:B), the plate was placed on a shaker plate at 37 °C for 30 min. After 30 min, the plate was placed on ice, and absorbance was measured at 562 nm on a SpectraMax M5 UV–Vis Spectrophotometer (Molecular Devices, San Jose, CA, USA). Percentage binding efficiency was calculated according to the following equation:

$$\mathrm{Binding}\;\mathrm{efficiency}=\frac{\left(\mathrm{initial}-\mathrm{supernatant}\right)}{\mathrm{initial}}\times 100\%$$

For the quantification of catalase mass in nanoparticles, 100 μL of catalase-loaded nanoparticles was combined with 50 μL of 1 M sodium hydroxide (ThermoFisher). The solution was vortexed for 2 s, spun down on a minicentrifuge, and then incubated at 37 °C for 30 min for base-catalyzed hydrolysis of the PLGA polymer to release all loaded catalase. 50 μL PBS was then added to neutralize the solution, and the solution was then measured according to the BCA assay kit.

### 2.3. Catalase Activity Assay

Catalase (catalase from bovine liver, Sigma) was used as a model enzyme due to its facile enzymatic activity measurement using a catalase spectrophotometric assay adapted from Beers and Sizer [25]. A pH 7.0 solution of 0.036% *w*/*w* H_2_O_2_ (Sigma) was prepared in 50 mM phosphate buffer with a 240 nm absorbance (A_240_) between 0.48 and 0.52. In an optically clear quartz cuvette (Hellma Analytics), 100 μL of catalase sample was added to 2.9 mL of H_2_O_2_ solution, mixed via pipetting, and A_240_ was measured at 2 s intervals for 3 min using a kinetic spectrometric reading on a SpectraMax M5 UV–Vis Spectrophotometer (Molecular Devices). The active units (AU) per mL of catalase solution (freely dissolved or encapsulated in nanoparticles) were calculated using the following equation:

$$\mathrm{Active}\;\mathrm{units}/\mathrm{mL}=\frac{\left(3.45\right)\left(\mathrm{dilution}\;\mathrm{factor}\right)}{(\mathrm{time})\times 0.1}$$

In the equation, 3.45 represents the decomposition of 3.45 μmol H_2_O_2_ during A_240_ decrease from 0.45 to 0.4, and 0.1 is the mL volume of sample added. This assay measures catalase activity even when the enzyme is encapsulated, due to the ability of H_2_O_2_ to diffuse throughout the polymer matrix [26]. Stock catalase had 2000–5000 AU/mg catalase.

### 2.4. Atomistic BSA/Ion Pairing (IP) Agent MD Simulations

GROMACS 2020.5 [27] was used to simulate all BSA/IP agent systems at 298.15 K, 1 bar and in a water/ion medium. BSA structure was taken from the RCSB protein database (PDB code 4F5S). Using an online MD preparation platform, PlayMolecule [28], BSA structure at pH 3.7 was extracted, where the predicted net charge was +78. AMBER99SB*-ILDNP forcefield [29] was used for BSA partial charge and topological parameters. GLYCAM-06j-1 forcefield [30] was used for DS topological parameters and the general amber forcefield (GAFF) [31,32] was used for SDS and TA topological parameters. An α-1,6 linked dodecamer was simulated for the BSA/DS simulations, due to its experimental molecular weight and high percentage of α-1,6 linkages [33]. Partial atomic charges for each IP agent were ascertained through the residual electrostatic potential fitting method [34], using the Hartree–Fock level with the 6–31G* basis set in Gaussian 0 9 [35]. A three-point (TIP3P) [36] explicit solvent model is used for water, while temperature control was achieved using the modified Berendsen Thermostat [37]; pressure control was achieved using the Parrinello–Rahman Barostat [38]. In order to maximize computational efficiency, the Hydrogen Mass Repartitioning method [39] was used to allow for a 4-femtosecond timestep. Calculation of backbone RMSD done using the built-in GROMACS function *gmx rms*. All production runs were 200 nanoseconds. All systems were packed within a cubic box with a side length of 15 nm using PACKMOL [40] and were charged neutralized with either potassium or chloride ions.

### 2.5. Fraction of Surface Residues with >95% Occupancy

Percent occupancy was calculated by counting the number of frames an IP agent was within 4 angstroms of a BSA amino acid surface amino acid (AA) divided by the total number of frames within the entire trajectory.

$$\%\;\mathrm{Occupancy}=\frac{\begin{array}{c} \mathrm{No}.\;\mathrm{of}\;\mathrm{frames}\;\mathrm{that}\;\mathrm{an}\;\mathrm{IP}\;\mathrm{molecule}\;\\ \mathrm{was}\;\mathrm{within}\;4\;Å\;\mathrm{of}\;a\;\mathrm{protein}\;\mathrm{residue} \end{array}}{\mathrm{Total}\;\mathrm{no}.\;\mathrm{of}\;\mathrm{frames}\;\mathrm{in}\;\mathrm{the}\;\mathrm{whole}\;\mathrm{trajectory}}\times 100$$

Once occupancy was extracted, residues with >95% occupancy were sub-selected from the total list of residues with a nonzero value of occupancy and were grouped into five categories: negative, positive, polar, hydrophobic and aromatic (Supplementary Materials Table S1). The number of residues in each grouping was then normalized by the total number of residues with >95% occupancy to allow for comparison.

$$\mathrm{Fraction}\;\mathrm{of}\;\mathrm{AA}\;\mathrm{with}\;>95\%\;\mathrm{occupancy}=\frac{\begin{array}{c} \mathrm{No}.\;\mathrm{of}\;\mathrm{residues}\;\mathrm{in}\;\mathrm{an}\;\mathrm{AA}\;\mathrm{grouping}\\ \mathrm{with}\;>95\%\;\mathrm{occupancy} \end{array}}{\mathrm{Total}\;\mathrm{no}.\;\mathrm{of}\;\mathrm{residues}\;\mathrm{with}\;>95\%\;\mathrm{occupancy}}$$

### 2.6. Nanoparticle Formulation

To formulate nanoparticles by S/O/W emulsion, 1 mL of 25 mg/mL PLGA45k-PEG5k (LA:GA 50:50, Akina, IN, USA) dissolved in dichloromethane (DCM, Fisher Scientific, Pittsburgh, PA, USA) was added to 1 mg lyophilized catalase HIP complexes. The mixture was emulsified with a Sonic Dismembrator Ultrasonic Processor (Fisher Scientific) using 20 kHz probe sonication at 30% amplitude with 1 s on:1 s off pulses for 30 s on. After adding 4 mL 3% cholic acid (Millipore Sigma) in DI water, the second sonication was performed at 20% amplitude with 1 s on:1 s off pulses for 30 s on. This emulsion was then poured into 25 mL beaker of 1% polysorbate 80 (P80, Millipore Sigma) and stirred for 3 h at 500 rpm to remove the organic solvent. Nanoparticles were collected and washed twice by ultracentrifugation with phosphate buffer at 100,000× *g* for 25 min. Finally, the nanoparticles were resuspended in 1 mL DI water. Nanoparticles were used immediately or stored at 4 °C for a short time.

To formulate nanoparticles by nanoprecipitation, 1 mg lyophilized catalase or BSA complexes were dissolved in 0.3 mL dimethyl sulfoxide (Millipore Sigma). 25 mg PLGA-PEG was dissolved in 0.7 mL acetone (Fisher Scientific). The two solutions were quickly vortexed together before being added dropwise into 25 mL 1% P80. The remainder of the procedure was as described above. For animal experiments, catalase and BSA nanoparticles were resuspended in sterile PBS.

### 2.7. Nanoparticle Characterization

Nanoparticle size and PDI were measured by dynamic light scattering. The ζ-potential was determined using a zeta potential analyzer (NanoSizer Zeta Series, Malvern Instruments, Malvern, UK). Samples were diluted to appropriate concentrations to obtain accurate measurements in 10 mM NaCl at room temperature, pH 7.4, as described previously [41].

Encapsulated catalase mass was measured by BCA assay kit and catalase activity was measured by activity assay, as described above. To determine catalase protection, nanoparticles were incubated in PBS with 0.2 wt% pronase (pronase from *Streptomyces griseus*, Sigma). Aliquots were collected at 0 h, 1 h, 2 h, 4 h, and 24 h, and immediately tested for catalase activity. Enzyme activities were calculated as the sample activity at a given timepoint divided by the initial sample activity at 0 h.

### 2.8. Animal Experiments and Ethics Statement

This study was performed in strict accordance with the recommendations in the Guide for the Care and Use of Laboratory Animals of the National Institutes of Health. All of the animals were handled according to approved Institutional Animal Care and Use Committee (IACUC) protocols (#4484-01) of the University of Washington, Seattle, WA, USA. The University of Washington has an approved Animal Welfare Assurance (#A3464-01) on file with the NIH Office of Laboratory Animal Welfare (OLAW), is registered with the United States Department of Agriculture (USDA, certificate #91-R-0001), and is accredited by AAALAC International. Every effort was made to minimize suffering. Sprague–Dawley female dams with sex-balanced litters (virus antibody-free CD^®^ (SD) IGS, Charles River Laboratories, Raleigh, NC, USA) were purchased and arrived when pups were postnatal day 5 (P5). The day of birth was defined as P0. Before and after the experiment, each dam and her pups were housed under standard conditions with an automatic 12 h light/dark cycle, a temperature range of 20–26 °C, and access to standard chow and autoclaved tap water ad libitum. The pups were checked for health daily.

### 2.9. Vannucci Model of Unilateral HI Injury in Neonatal Rats and Drug Administration

Although the bulk of historical studies have been conducted in P7 animals, the P10 Vannucci model was chosen for this study because a number of more recent preclinical studies suggest that the P10–11 rat more closely mimics the brain maturation of the term infant [42]. In the P10 Vannucci model, the cerebral structures most likely to be damaged are the hippocampus, cortex, striatum, and thalamus, depending on the severity of insult [43]. These patterns of injury, as well as the response to TH, are broadly comparable to those seen in infants with HIE [42].

On P10, pups were separated from their dams, weighed and sexed, and randomized to experimental groups. Anesthesia with isoflurane (3–5%) was given in 100% O_2_ via a nose cone, under a dissecting microscope. The left carotid artery was identified and ligated. Pups were maintained in a temperature-controlled water bath before and after undergoing unilateral ligation of the left carotid artery. After all the animals recovered from anesthesia, they returned to the dams for a minimum of 30 min before placement in a hypoxic chamber in a temperature-controlled water bath. Once rectal temperature in a sentinel animal was stable at 36 °C for 5 min, the chamber was sealed and 8% O_2_ (92% N_2_) administered at a rate of 2.5 L/min. Once the oxygen concentration within the chamber reached 8%, hypoxia was maintained for approximately 2 h or until 10% mortality was reached. The end of hypoxia marked the end of the insult (i.e., 0 h timepoint). Normal nesting rectal temperature at P10 would be expected to range from ~35–37 degrees Celsius [44]. As expected [45], all animals were relatively hypothermic immediately after hypoxia (Figure S1A). No significant differences were found between groups, and all animals maintained normal temperature during the temperature management period after injury, which is used to ensure that any therapeutic effects are not confounded by temperature differences between groups. The pups were returned to the dam for 30 min, after which the first dose of treatment was administered and the pups’ temperatures were monitored for 5 h to ensure normothermia. The median (IQR) temperature during this period was 36.6 °C (36.4–36.7 °C), 36.7 °C (36.4–37.0 °C), and 36.6 °C (36.4–36.8 °C) for the saline, blank nanoparticle, and catalase nanoparticle groups, respectively (Figure S1B). No differences in rectal temperature were seen between groups at any time.

Treatments were administered intraperitoneally at 30 min, 24 h, and 48 h after injury. A total of 58 pups (30 males, 28 females) were randomized into three separate treatment groups: saline (12 males, 11 females), blank nanoparticles (9 males, 7 females), and catalase nanoparticles (9 males, 10 females). Catalase nanoparticles were dosed at 3300 AU/kg. An equivalent polymer and protein mass was delivered with the blank formulation. Dosage and timing were based on previous investigation of the therapeutic window for pharmacological agents in the Vannucci model [15,46].

### 2.10. Gross Injury Scoring and Area Loss

72 h after injury, animals received an overdose of pentobarbital before transcardiac perfusion with 0.9% saline. Immediately following brain extraction, a photo of each whole brain was taken for gross injury scoring. The brain was then sliced into 3 mm thick slices at approximately the level of the hippocampus and thalamus. These sections contain the cerebral structures most likely to be damaged in the P10 Vannucci model and in infants with HIE [42]. Slices were incubated in prewarmed 2,3,5-triphenyltetrazolium chloride (TTC, Fisher Scientific) for 10 min at 37 °C. The slices were then fixed in 10% neutral buffered formalin for 24 h before being imaged for area loss measurement.

Both gross injury and area loss scoring was conducted by two independent individuals who were blinded to group allocation. Gross brain injury in the hemisphere ipsilateral to ligation was assessed on a five-point ordinal scale (0–4) as follows: 0 = no injury, 1 = mild injury with <25% lesion of ipsilateral hemisphere, 2 = 25–50% lesion, 3 = 51–75%, and 4 = ≥75% injury, as previously described [47]. Area loss was quantified by measuring the area of healthy tissue in the ipsilateral hemisphere normalized to the contralateral hemisphere, according to the following equation:

$$\%\;\mathrm{Area}\;\mathrm{Loss}=\left(1-\frac{\mathrm{ipsilateral}\;\mathrm{area}}{\mathrm{contralateral}\;\mathrm{area}}\right)\times 100\%$$

### 2.11. Immunofluorescence and Confocal Imaging

PLGA-PEG uptake in the brain and microglial morphology was evaluated by placing freshly extracted brains in a formalin-to-30% sucrose gradient and then sectioning on a Leica cryostat into 30 µm sections. For microglia, a primary antibody solution (1:250 rabbit anti-Iba1, Wako) was prepared in 1xPBS containing 1% Triton-X (Sigma) and 3% normal goat serum (Sigma) and was added to tissue sections for 4 h in a humidified chamber at room temperature. Sections were washed twice in 1xPBS. A secondary antibody solution was prepared in 1xPBS and 1% Triton-X and added to tissue sections for 2 h. For neurons, a pre-conjugated antibody solution (1:500 anti-NeuN AlexaFluor 488, Abcam, Cambridge, UK) was prepared in 1xPBS containing 1% Triton-X (Sigma) and added to tissue sections for 6 h in a humidified chamber at room temperature. Sections were washed twice in 1xPBS and then stained with 1:10,000 DAPI for 10 min (Invitrogen, Waltham, MA, USA). Slides were washed and dried for 30 min in the dark. Mounting medium (Dako, Agilent Technologies, Santa Clara, CA, USA) was added to each slide and a glass coverslip placed on top. Slides were stored at 4 °C until imaged on an A1 confocal microscope (Nikon Instruments, Melville, NY, USA) and at 20 °C for long-term storage.

### 2.12. Statistical Analysis

All statistical analyses were carried out in GraphPad Prism (GraphPad Software Inc, Version 8.4.0, San Diego, CA, USA). For analysis of complexation efficiency and nanoparticle catalase loading, the unpaired *t*-test with Welch’s correction was used to test for significance. Injury data was summarized as a median with IQR. Total area loss and gross injury scores were compared by the two-tailed Wilcoxon–Mann–Whitney U-test. Data with a *p* value <0.05 were considered statistically significant.

## 3. Results

### 3.1. Effect of Ion-Pairing Agent, Molar Ratio, pH, and Buffer Ion on Complexation Efficiency

**Figure 1:**
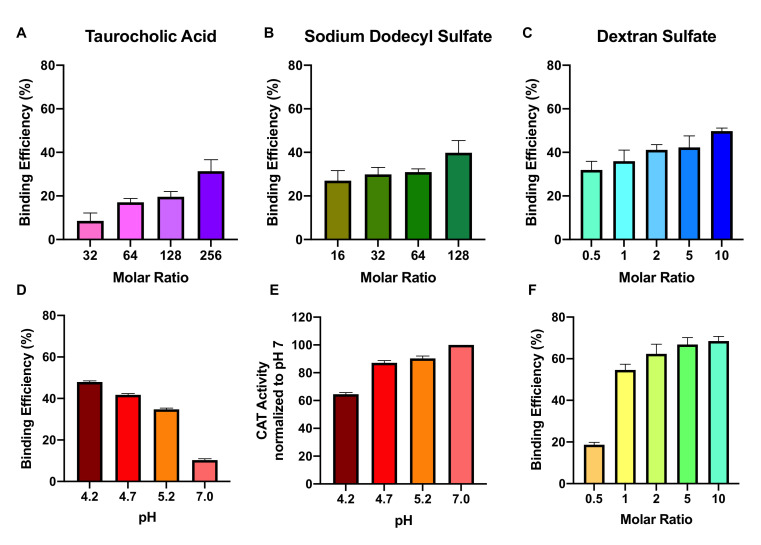
Characterization of DS-catalase complexation under various formulation conditions. (**A**) TA, (**B**) SDS, and (**C**) DS demonstrated an increasing trend of catalase binding efficiency with respect to increasing molar ratio. For DS-CAT complexes, more acidic pHs are associated with (**D**) increased binding efficiency but (**E**) increased loss of activity. (**F**) Binding efficiency in citrate buffer increases with increasing molar ratio. Values are represented as mean ± SD (*n* = 3).

We first optimized the HIP complexation of catalase by investigating three common sulfated ion-pairing agents, taurocholic acid (TA), sodium dodecyl sulfate (SDS), and dextran sulfate (DS), across a range of molar ratios. Phosphate buffer at pH 4.7 was used for all initial experiments. For each ion-pairing agent, catalase binding efficiency increased with increasing molar ratio. Catalase incubation with TA at ion-to-protein molar ratios of 32, 64, 128, and 256 resulted in 8.6, 16, 20, and 31% binding efficiencies, respectively (Figure 1A). For SDS, molar ratios 16, 32, 64, and 128 led to 27, 30, 31, and 40% binding efficiencies (Figure 1B), and molar ratios of DS at 0.5, 1, 2, 5, and 10 led to 32, 36, 41, 42, and 50% binding efficiencies, respectively (Figure 1C).

To further optimize complexation, we investigated the role of pH on catalase binding efficiency using DS as the ion-pairing agent. Lower pHs led to greater binding efficiency: at pH 4.2, 4.7, 5.2, and 7.0, we observed binding efficiencies of 48, 42, 35, and 10%, respectively (Figure 1D). However, lower pH was also associated with higher catalase degradation, as measured by a loss of activity. When normalized to catalase activity in phosphate buffer at pH 7, catalase at pH 4.2, 4.7, and 5.2 retained 65, 87, and 90% of its activity, respectively (Figure 1E).

As phosphate has a reduced buffering capacity at acidic pHs, we therefore investigated DS-catalase complexation with citrate buffer, which has a working range of pH 3.0–6.2. At ion-to-protein molar ratios of 0.5, 1, 2, 5, and 10, we observed binding efficiencies of 19, 55, 62, 67, and 68%, respectively (Figure 1F). In comparison to phosphate buffer, citrate buffer achieved significantly higher binding efficiencies at every molar ratio above 0.5 (*p* < 0.05 for all).

### 3.2. Molecular Scale Features of Protein-Ion Complexes

In order to understand any potential differences in the behavior of DS, SDS and TA when binding to proteins, we performed MD simulations to evaluate the structure and dynamics of protein-ion complexes. As a model protein, we used BSA instead of catalase owing to its smaller size, which enables significantly longer simulation times. BSA is well matched to catalase, having a similar profile of surface amino acid residues as shown in Supplementary Materials Figure S2. As such, it is reasonable to expect that the nature and extent of protein-ion interactions we obtain from the BSA/ion-pairing agent simulations will provide useful insight to the behavior of other proteins with a similar surface profile.

**Figure 2:**
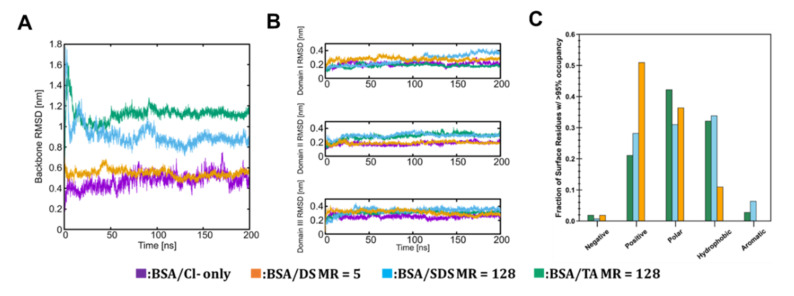
Characterization of BSA structure and IP agent interactions with surface amino acids. (**A**) BSA backbone RMSD (in nanometers [nm]) vs. time (in nanoseconds [ns]), (**B**) Backbone RMSD of each BSA domain vs. time, (**C**) Fraction of surface residues with >95% occupancy vs. residue grouping.

Following completion of the MD simulations, we analyzed the structure of BSA and related conformational changes. We also analyzed the chemical interactions between ions and different types of residues at mole ratios of 128 (SDS, TA) and 5 (DS), which were selected to roughly control for a consistent number of anionic sulfate groups between DS (120 total) and SDS and TA (128 total). The MD simulation analyses for these systems are shown in Figure 2. As previously noted by Baler et al. [48], we expect BSA at pH 3.7 to undergo a conformational rearrangement of tertiary structure even on the timescale of MD simulation (usually hundreds of nanoseconds). Figure 2A shows BSA backbone root mean squared deviation (RMSD) from its crystal structure as a function of simulation time for the three ion-paired systems as well as a control system with only Cl- present for charge neuralization. The level of conformational change in the control and DS system (RMSD ~0.5 nm at 200 ns) corresponds well to the expected structure of BSA in the N-isoform. In contrast, BSA conformational changes in the SDS and TA systems (RMSD ~0.9 nm and ~1.1 nm at 200 ns) indicate that the protein is transitioning from the N to the F-isoform. This suggests that DS complexation is able to retain BSA’s native state, unlike SDS and TA complexation. Snapshots of the final structures of each of the simulations are provided in Supplementary Materials Figure S3. The individual domains of BSA do not undergo any significant unfolding for any of the systems (Figure 2B). Finally, we observe that SDS and TA behave similarly from the point of view of the dominant chemical interactions on the BSA surface (Figure 2C), showing significant interactions with hydrophobic and aromatic residues. In contrast, DS has comparatively very few interactions with these residue types.

### 3.3. Effect of Nanoparticle Formulation Method on Catalase Loading and Protection

We next incorporated DS-catalase complexes into PLGA-PEG nanoparticles using previously published methods for nanoprecipitation and solid/oil/water (S/O/W) emulsion [20,22]. Dynamic light scattering results are summarized in Table 1. By nanoprecipitation, catalase-loaded nanoparticles had an average diameter of 115.8 nm, polydispersity index (PDI) of 0.17, and ζ-potential of −2.3 mV. By emulsion, catalase-loaded nanoparticles had an average diameter of 125.4 nm, PDI of 0.25, and ζ-potential of −5.6 mV. Table 1 also includes size and surface charge characterization of a blank formulation, used as a control for in vivo studies described in the following section. For the control nanoparticles, DS was complexed with BSA at a molar ratio of 5 and pH 3.8.

**Figure 3:**
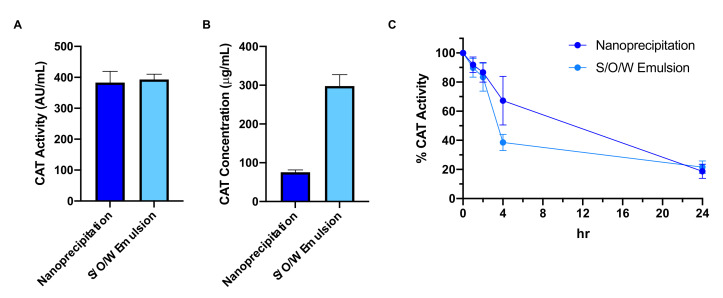
Catalase loading and protection in PLGA-PEG nanoparticles by formulation method. (**A**) Nanoprecipitation and emulsion nanoparticles achieve non-significantly different catalase loading by activity, but (**B**) nanoprecipitation nanoparticles have significantly lower catalase loading by mass (*p* = 0.0003). (**C**) Both methods result in retention of catalase activity over 24 h in 0.2% pronase solution. Values are represented as mean ± SD (*n* = 4 for A and B; *n* = 3 for C).

To compare the two catalase nanoparticle formulations, we assessed catalase loading by activity and mass. In terms of catalase activity, both formulations achieved similar loading: nanoprecipitation particles had a mean ± standard deviation 383 ± 73 active units (AU) of catalase per mL nanoparticles, while emulsion particles had 393 ± 34 AU/mL (Figure 3A). In terms of catalase loading by mass, however, nanoprecipitation particles (76 ± 12 μg/mL) had significantly less catalase than emulsion particles (298 ± 59 μg/mL, *p* = 0.0003) (Figure 3B). Together, these two results indicate higher catalase deactivation by the emulsion process. Catalase activity after nanoprecipitation averages around 5000 ± 960 AU/mg (as supplied by Sigma); emulsion results in catalase activity of 1320 ± 110 AU/mg. The emulsion process therefore corresponds to 74% (± 2%) deactivation of catalase.

Next, we assessed nanoparticle protection of catalase in biological media. To mimic degradative serum conditions in vivo, we incubated nanoparticles in a 0.2% pronase solution and measured catalase activity at 0, 1, 2, 4, and 24 h (Figure 3C). Activity was normalized to the 0h timepoint. No significant differences were observed between formulations at each timepoint, and 20% of initial catalase activity was retained by both formulations at the end of the 24 h experimental window.

### 3.4. Effect of Catalase-Loaded Nanoparticles on Brain Injury Severity in Neonatal Rats

**Figure 4:**
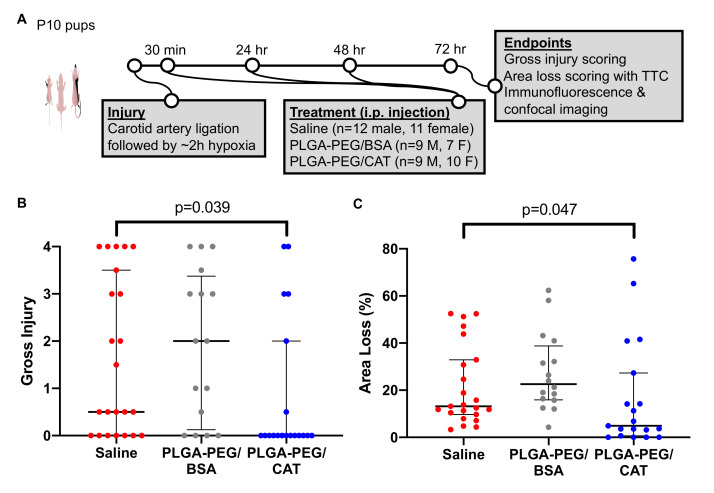
Global brain injury is significantly reduced by treatment with catalase-loaded nanoparticles. (**A**) Rats were injured at P10, received treatment 30 min, 24 h, and 48 h after injury, and were sacrificed at 72 h for endpoint analysis. (**B**) Median (IQR) gross injury scores in the saline, blank nanoparticle, and catalase (CAT) nanoparticle groups are 0.5 (0–3), 2 (0–3.5), and 0 (0–2). (**C**) Median (IQR) area loss measurements in the saline, blank nanoparticle, and catalase nanoparticle groups are 13% (10–31%), 23% (16–41%), and 4.9% (0.61–27%). For both assessments, treatment with catalase is significantly neuroprotective compared to saline (*p* = 0.039 and *p* = 0.047, respectively) while blank nanoparticles have no significant effect.

Given the significant catalase deactivation by emulsion, we pursued nanoparticles formulated by nanoprecipitation for in vivo investigation. We assessed the efficacy of catalase-loaded nanoparticles in the Vannucci model of HIE with P10 rat pups. Treatments of saline, blank nanoparticles (3300 AU BSA/kg), or catalase nanoparticles (3300 AU catalase/kg) were administered intraperitoneally 30 min, 24 h, and 48 h after injury (Figure 4A). At the 72 h endpoint, we found a significant reduction in gross injury scores after catalase-loaded nanoparticle treatment where median (interquartile-range, IQR) score was 0 (0–2) compared to saline treatment (0.5, 0–3; *p* = 0.039) (Figure 4B). Treatment with blank nanoparticles (2, 0–3.5) did not have a significant effect on gross injury.

Total area loss measurements supported these results (Figure 4C). The median (IQR) injury after saline treatment was 13% (10–31%), which was significantly reduced in catalase nanoparticle-treated pups to 4.9% (0.61–27%; *p* = 0.047) but was not significantly affected by treatment with blank nanoparticles (23%, 16–41%). Representative gross injury and area loss images from the median pup in each group are shown in Supplementary Materials Figure S4.

**Figure 5:**
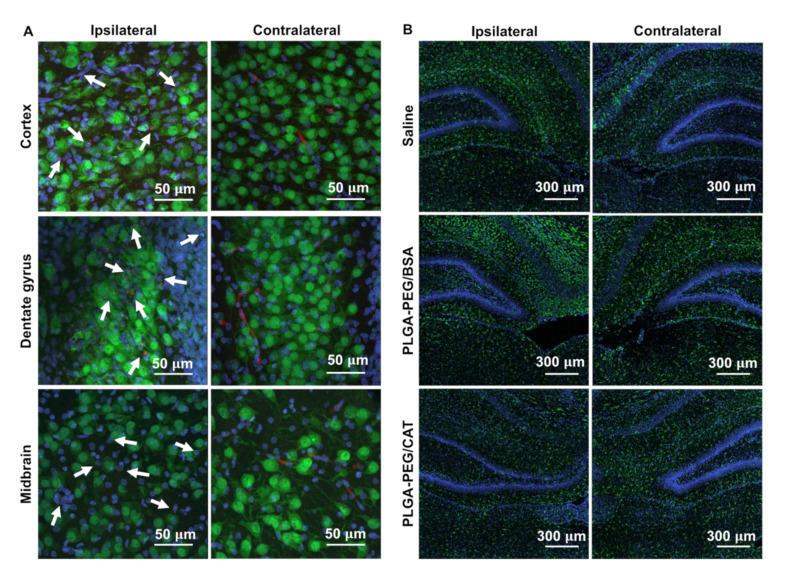
Nanoparticle distribution and microglial response to treatment in the ipsilateral hemisphere. (**A**) PLGA-PEG nanoparticles (red) are observed in the cortex, dentate gyrus, and midbrain of the injured hemisphere, but appear trapped in the vasculature of the contralateral hemisphere. Neurons (green) and cell nuclei (blue) are also shown. Scale bars: 50 µm. (**B**) Microglia (green) have increased number and density in the ipsilateral compared to contralateral hemisphere in saline- and blank nanoparticle-treated pups. After catalase nanoparticle treatment, microglia number and density appear similar between hemispheres. Scale bars: 300 µm. All cell nuclei are shown in blue.

Using confocal imaging, we next confirmed the distribution of nanoparticles in injured brain tissue. In the contralateral hemisphere, nanoparticles were observed in blood vessel-like structures, consistent with normal blood-brain barrier function. In contrast, PLGA-PEG nanoparticles were widely distributed through the ipsilateral hemisphere, including the cortex, dentate gyrus, and midbrain regions (Figure 5A). Imaging of microglia in the hippocampal region demonstrated a cell-level response to PLGA-PEG/CAT nanoparticle treatment. Microglia were higher in number and density in the ipsilateral hemisphere compared to the contralateral hemisphere in both the saline and PLGA-PEG/BSA control groups. After catalase-loaded nanoparticle treatment, microglia number and density appear more consistent between hemispheres, supporting improvement of neuroinflammation (Figure 5B).

## 4. Discussion

In this study, we used HIP to increase the lipophilicity of catalase, promoting its encapsulation in PLGA-PEG nanoparticles for therapeutic application. We first optimized catalase binding efficiency across ion-pairing agents, molar ratios, pH, and buffer ion. For all variations, catalase complexed quickly with TA, SDS, and DS; the solution immediately turned cloudy with insoluble precipitates. This is in good agreement with previous studies investigating these ion-pairing agents with a number of proteins including lysozyme, conalbumin, insulin, and ovalbumin [49,50,51]. Catalase, however, is the largest by far among these enzymes; the large molecular weight may explain the lower binding efficiencies observed in our study (<50% in phosphate buffer, <70% in citrate buffer) compared to previous work with other proteins (>90%) [20,22]. Hydrophobic and ionic interactions may be sterically limited in a large tetramer like catalase with complex tertiary and quaternary structure [19,52]. Despite this, we still observed successful complexation, encouraging further study of HIP with large enzymes.

HIP complexation is driven by electrostatic interactions. At pH 4.7, catalase (pI 5.4) is positively charged while the ion-pairing agents are negatively charged due to their sulfate groups (pKa < 2). The ion-to-protein charge ratio was approximately 1:1 for experimental trials TA 128, SDS 128, and DS 5, where binding efficiency was 20, 31, and 42% respectively. Lower binding efficiency with TA and SDS may be attributable to their higher hydrophobicity and lower charge density compared to DS [20]. Molecular simulations indicate that DS-bound BSA behaves more similarly to the native protein in terms of structural evolution on timescales of 100s of nanoseconds and, in contrast to SDS and TA, limits strong interactions of hydrophobic/aromatic residues, which may help to stabilize the structure. At pH 4.2, catalase becomes further positively charged and we observed higher binding efficiency with DS 5 (48%). The cost is a loss of catalase function, in alignment with previous work describing deactivation of catalase below pH 4 [53]. In contrast, when catalase is more neutrally charged and fully active at pH 7, hydrophobic interaction-driven binding resulted in only 10% efficiency. Our study also highlights the importance of buffer stability at acidic pHs; using citrate buffer (stable between pH 3.2–6.0), we observed significantly higher binding efficiencies at most DS molar ratios compared to phosphate buffer. Additionally, we could not produce BSA-DS complexes in phosphate buffer at molar ratios and pHs which easily formed complexes in citrate buffer. Although phosphate buffer has been previously studied in HIP complexation [22], our study supports the use of citrate buffer for complexation with large proteins.

We found improved binding efficiency by increasing the molar ratio for each ion-pairing agent. Previous reports have found a limit to this behavior, where further molar ratio increments past a critical point result in decreased binding [20,22]. The proposed mechanism is that excess ion-pairing agents form micelles which provide a hydrophobic environment in which complexes can be solubilized or dissociate [54]. A large molecular-weight protein like catalase may require higher molar ratios to exhibit this behavior, or the large protein may interfere with micellization. Further work must be done to fully understand the limits of molar ratio to increase catalase binding efficiency. Other parameters, including increased incubation time [20] or larger molecular weight ion-pairing agents [55], may also be worth investigating for improved catalase binding efficiency.

The water-insoluble catalase complexes were then used to improve enzyme loading in PLGA-PEG nanoparticles. Nanoparticle loading of HIP complexes has been previously demonstrated [55], although never with complexes of large enzymes. The conventional method for nanoparticle enzyme encapsulation, water in oil in water (W/O/W) double emulsion, depends on protein partitioning into an organic polymer matrix during a first emulsion. However, hydrophilic protein molecules rapidly penetrate to the external aqueous phase during the second emulsion, leading to poor encapsulation [19,56]. Additionally, high-energy sonication with PEG and DCM may result in byproducts that exacerbate oxidative stress in models of neurological injury [57]. In contrast, the nanoprecipitation technique involves low-energy mixing of organic and aqueous phases, but results in poor catalase encapsulation as evidenced by rapid loss of catalase activity in degradative conditions [57]. We hypothesized that hydrophobic catalase-DS complexes would demonstrate improved partitioning with PLGA-PEG and improved catalase protection in degradative conditions. Our results supported this hypothesis. Both S/O/W and nanoprecipitation particles retained 20% of their initial catalase activity over 24 h in a pronase solution. In a previous study, catalase-loaded W/O/W and nanoprecipitation nanoparticles only retained 6.1% and 5.4% activity, respectively, over 24 h [57]. Variability at earlier timepoints in this experimental window (0–4 h) may be attributed to nanoscale characteristics such as uneven catalase distribution between nanoparticles, which must be further investigated in future work. We also showed a pronounced protein deactivation effect with the emulsion method: while both S/O/W and nanoprecipitation particles had similar activity (400 AU/mL), emulsion particles loaded approximately fourfold higher catalase mass. We therefore proceeded to in vivo evaluation with complexed-catalase-loaded nanoparticles formulated by nanoprecipitation.

In the P10 Vannucci rat model of neonatal HIE, we observed a robust neuroprotective effect in the catalase-loaded nanoparticle treatment group compared to the saline control group, as evidenced by gross injury and area loss scoring as well as microglial morphology. No neuroprotective effect was observed in the nanoparticle control group using BSA-DS complexes. Catalase scavenging of hydrogen peroxide may combat HIE progression in multiple ways: it alleviates ROS burden in the injured brain [58], serves as an alternate source of oxygen in hypoxic tissue [59], and can mitigate long-term inflammatory processes [60]. Nanoparticle delivery of catalase has previously proved efficacious after MCAO and traumatic brain injury in adult mice [59,60] and thromboembolic stroke in adult rats [58]. Compared to adults, neonates have relatively immature antioxidant defenses and a reduced ability to regenerate antioxidants under HI conditions [61,62]. To our knowledge, this study is the first investigation of catalase to provide antioxidant relief after neonatal brain injury. Coadministration of catalase with superoxide dismutase, another antioxidant enzyme which converts oxygen radicals to hydrogen peroxide, may further enhance neuroprotection in this model and is a focus of future work.

One limitation of our in vivo study is that overall injury severity is relatively low. Gross injury scores from the saline group show a bimodal injury distribution which is characteristic of this model. However, our median area loss was only 13%, compared to around 25–35% achieved in similar, previous studies [43,63,64,65]. Very large group sizes may be required to address this limitation and determine true effect sizes, which is a focus of ongoing work. Increased animal numbers would also enable the assessment of sex-based differences in response to treatment. Significant changes in outcome based on sex have been observed in both preclinical models and in clinical settings [66,67], and males may potentially display decreased antioxidant defenses compared to females [68,69]. Our results encourage the further study of catalase-loaded nanoparticles as a pharmaceutical intervention in perinatal brain injury.

## 5. Conclusions

Neonatal hypoxic-ischemic brain injury often results in a lifelong burden of disease, and strategies to better treat this condition are needed. The antioxidant enzyme catalase is a promising therapeutic which would benefit from PLGA-PEG nanoparticle delivery for improved protection from serum proteases and improved delivery into the brain parenchyma. However, large hydrophilic enzymes do not easily partition into the polymer nanoparticle matrix. We demonstrated that hydrophobic ion-pairing could be used to formulate DS-catalase complexes with 68% binding efficacy. MD simulations supported that DS binding did not interfere with the native secondary or tertiary protein structure. The DS-catalase complexes were then used to develop catalase-loaded nanoparticles with high catalase activity and protection of enzyme activity for at least 24 h in degradative conditions. Finally, catalase-loaded nanoparticles were found to be significantly neuroprotective in the P10 Vannucci model, resulting in reduced injury scores as well as improved microglial morphology compared to saline and blank nanoparticle controls. Catalase-loaded PLGA-PEG nanoparticles are therefore a promising intervention for further research on the treatment of neonatal brain injury.

## 6. Patents

A provisional patent based on this work has been filed: “Formulation and efficacy of enzyme-loaded polymeric nanoparticles.” Provisional Patent Application 63/221,827 filed 7/14/2021.

## Figures and Tables

**Table 1:** Nanoparticles were characterized in terms of hydrodynamic diameter, mean surface charge (ζ-potential), and the PDI by dynamic light scattering at 25 °C and pH 7.2 in 10 mM NaCl. All values are reported as mean ± standard error of the mean (SEM) (n = 3).

Protein	Formulation Method	Number Mean ± SEM (nm)	PDI	ζ-Potential ± SEM (mV)
Catalase	Nanoprecipitation	115.8 ± 1.9	0.17	−2.3 ± 0.2
Catalase	S/O/W emulsion	125.4 ± 5.2	0.25	−5.6 ± 0.4
Bovine serum albumin	Nanoprecipitation	106.5 ± 5.4	0.13	−2.6 ± 0.1

## Data Availability

The data presented in this study are available from the corresponding author on request.

## Supplementary Materials

The following are available online at https://www.mdpi.com/article/10.3390/pharmaceutics13081131/s1, Table S1: Amino acid grouping, Figure S1: Rectal temperature measurements of P10 rats; Figure S2: Surface amino acid composition of BSA, Figure S3: End of trajectory snapshots for ion-pairing agents, Figure S4: Representative gross injury and area loss images.
