# Supplementary material for: Formulation and Efficacy of Catalase-Loaded Nanoparticles for the Treatment of Neonatal Hypoxic-Ischemic Encephalopathy
Source: Pharmaceutics. 2021 Jul 23;13(8):1131. doi: 10.3390/pharmaceutics13081131 (PMC8400001; doi:10.3390/pharmaceutics13081131)
Supplement: Supplementary file 1 [file pharmaceutics-13-01131-s001.zip › pharmaceutics-1280023-supplementary.pdf]

pharmaceutics

Supplementary Materials: Formulation and Efficacy of Catalase-Loaded Nanoparticles for the Treatment of Neonatal Hypoxic-Ischemic Encephalopathy

Andrea Joseph [1], Chris W. Nyambura [1], Danielle Bondurant [1], Kylie Corry [2], Denise Beebout [1], Thomas R. Wood [2], Jim Pfaendtner [1] and Elizabeth Nance [1,*]

Table S1. Amino Acid categories for residues with >95% occupancy.

Amino Acid Grouping	Residue (Label)
Negative	Aspartic Acid (ASP)
	Glutamic Acid (GLU)
Positive	Lysine (LYS)
	Arginine (ARG)
	Fully Protonated Histidine (HIP)
Polar	Asparagine (ASN)
	Cysteine (CYS)
	SS-bonded Cysteine (CYX)
	Protonated Glutamic Acid (GLH)
	Protonated Aspartic Acid (ASH)
	Glutamine (GLN)
	Threonine (THR)
	Serine (SER)
	Glycine (GLY)
	Histidine (HIS or HIE or HID)
Hydrophobic	Alanine (ALA)
	Isoleucine (ILE)
	Leucine (LEU)
	Valine (VAL)
	Proline (PRO)
	Phenylalanine (PHE)
	Tryptophan (TRP)
	Methionine (MET)
	Tyrosine (TYR)
Aromatic	Phenylalanine (PHE)
	Tryptophan (TRP)
	Tyrosine (TYR)
	Histidine (HIS or HID or HIE)

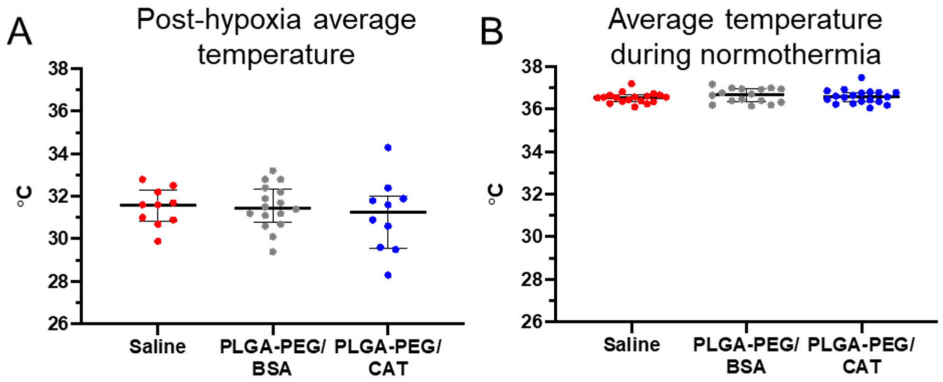

Figure S1. Rectal temperature of P10 rats (**A**) immediately after hypothermia and (**B**) during normothermia.

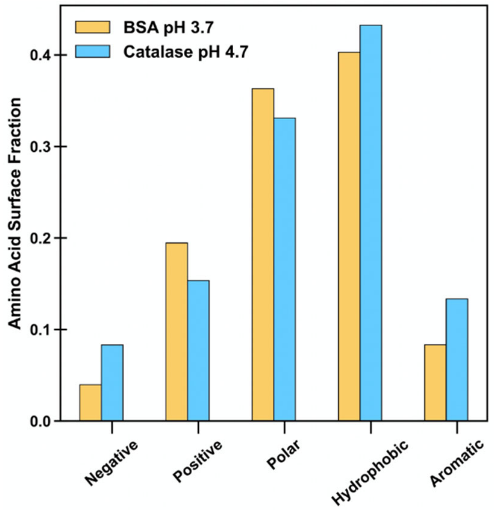

Figure S2. Surface amino acid composition of BSA at pH 3.7 in its native state, compared to catalase at pH 4.7.

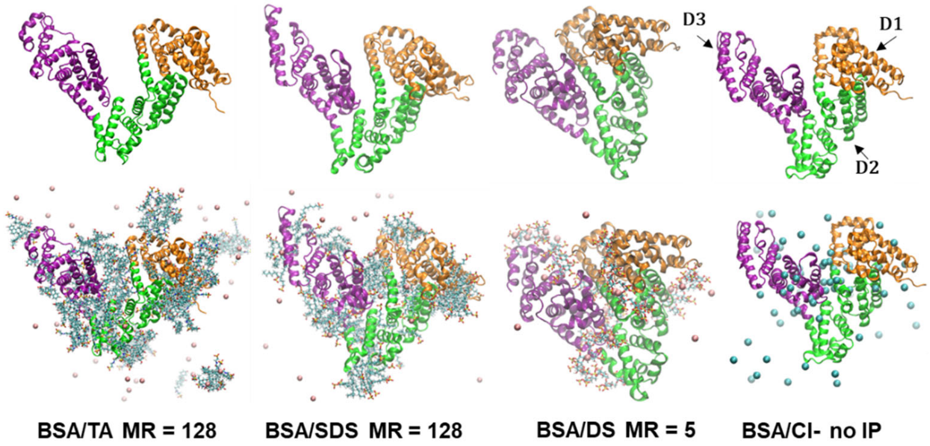

Figure S3. End of trajectory snapshots (t = 200 ns) of BSA without (top row) and with (bottom row) ion-pairing agents and neutralizing ions. Chlorine ions are represented by cyan particles and potassium ions are represented by brown particles. Domain I (D1) is in orange, Domain II (D2) is in green and Domain III (D3) is in purple.

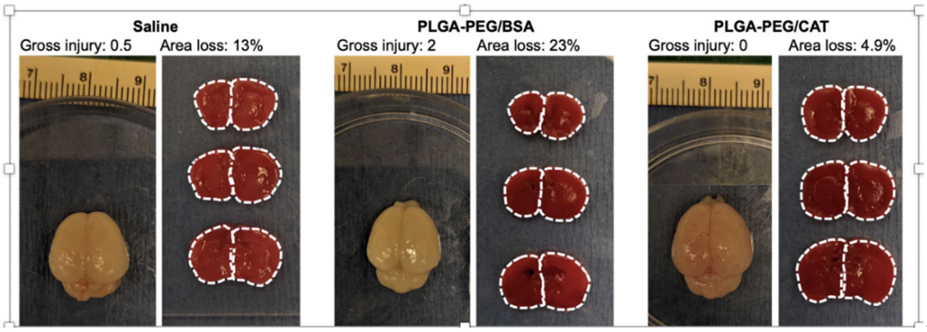

Figure S4. Representative gross injury and area loss images from each group. In the saline and PLGA-PEG/BSA groups, the left hemisphere (left side of each slice) is noticeably smaller than the right hemisphere.
